# Engineered Magnetic-Functionalized Carbon Xerogels for Sustainable Arsenic Removal: Bridging Adsorption Efficiency with Regenerability

**DOI:** 10.3390/gels11050323

**Published:** 2025-04-26

**Authors:** Sasirot Khamkure, Prócoro Gamero-Melo, Audberto Reyes-Rosas, Alejandro Zermeño-González, José Luis Álvarez-Cruz, Elim Albiter Escobar, Gabriela Eleonora Moeller-Chávez, Victoria Bustos-Terrones

**Affiliations:** 1 Departmento de Irrigación y Drenaje, Secihti- Universidad Autónoma Agraria Antonio Narro, Saltillo 25315, Mexico; 2 Sustainability of Natural Resources and Energy, Centro de Investigación y de Estudios Avanzados del IPN, Unidad Saltillo, Ramos Arizpe 25900, Mexico; procoro.gamero@cinvestav.edu.mx; 3 Departmento de Biosciencia y Agrotecnología, Centro de Investigación en Química Aplicada, Saltillo 25294, Mexico; audberto.reyes@ciqa.edu.mx; 4 Departmento de Irrigación y Drenaje, Universidad Autónoma Agraria Antonio Narro, Calzada Antonio Narro, Saltillo 25315, Mexico; azermeno@uaaan.edu.mx; 5 Laboratorio de Catálisis y Materiales, Escuela Superior de Ingeniería Química e Industrias Extractivas —Instituto Politécnico Nacional, Zacatenco, Mexico City 07738, Mexico; jalvarezc@ipn.mx (J.L.Á.-C.); ealbitere@ipn.mx (E.A.E.); 6 Laboratorio de Investigación en Ingeniería Ambiental y Sustentabilidad, Universidad Politécnica del Estado de Morelos, Jiutepec 62574, Mexico; gmoeller@upemor.edu.mx

**Keywords:** arsenic, functionalized xerogels, response surface methodology, sol–gel, regeneration

## Abstract

This study developed iron-oxide-functionalized carbon xerogels for enhanced arsenic adsorption to mitigate global water contamination. The composites were synthesized by integrating magnetite nanoparticles (15–20 nm) into a resorcinol–formaldehyde matrix via sol–gel polycondensation, followed by controlled pyrolysis. Key parameters—magnetite/resorcinol ratios (0.03–0.07), carbonization conditions (temperature, heating rate, duration), and H_2_O_2_-induced surface modification—were optimized to maximize adsorption performance. Characterization (SEM/EDX, XRD, FTIR, BET, TEM) confirmed uniform magnetite dispersion (~5 wt%) and revealed that pyrolysis at 850 °C enhanced porosity (378.8 m^2^/g surface area) and refined surface chemistry. Adsorption kinetics followed Elovich (R^2^ = 0.9396) and Power Function (R^2^ = 0.9443) models, indicating chemisorption dominance. Response Surface Methodology optimized desorption parameters using a Central Composite Design with three factors and two center points with repetition. A kinetic study of As(V) desorption from carbon xerogels was conducted, yielding optimal conditions: 1.0 M KOH, 160 rpm agitation, and 90 min contact time. The adsorbent retained >88% regeneration efficiency over four cycles, demonstrating robust reusability. Synergistic effects of magnetite incorporation, tailored pyrolysis, and H_2_O_2_ modification significantly improved arsenic selectivity and capacity in complex matrices, while enabling magnetic recovery.

## 1. Introduction

The contamination of aquatic environments with arsenic, a potent carcinogen, threatens millions globally, with chronic exposure linked to cancers, cardiovascular diseases, and neurological disorders [1]. Adsorption remains a cornerstone technology for arsenic removal due to its operational simplicity and adaptability [2]. However, the economic and environmental sustainability of adsorption-based water treatment hinges on the regenerability and reusability of adsorbents—a challenge often overlooked in favor of adsorption efficiency alone [3].

Magnetic nanoparticles (Fe_3_O_4_ NPs) offer a promising solution for contaminant recovery via magnetic separation, enhancing adsorbent recyclability [4]. Conventional co-precipitation methods, while cost-effective and scalable [5], raise ecological concerns due to nanoparticle toxicity [6,7]. To mitigate these risks, this study proposes magnetic-functionalized carbon xerogels (XMCs)—a hybrid material combining the adsorptive prowess of carbon xerogels with the magnetic functionality of Fe_3_O_4_ nanoparticles.

Carbon xerogels are renowned for their tunable porosity, high surface area, and chemical stability, making them ideal for environmental applications [8,9]. Their synthesis via sol–gel polymerization allows precise control over microstructure, while post-treatments like activation and doping further enhance functionality [10]. Recent advances in heteroatom doping and metal incorporation have expanded their utility in catalysis and adsorption [11]. However, their nanostructure’s sensitivity to synthesis parameters (e.g., pH, catalyst concentration) demands meticulous optimization [12].

By embedding magnetite nanoparticles (Fe_3_O_4_ NPs) into carbon xerogels, this study addresses three critical challenges. (1) Minimizing environmental release of nanomaterials [13]. (2) Enhancing arsenic removal through conventional co-precipitation methods, where the specific stoichiometric ratios of Fe^3+^ and Fe^2+^ are critical for forming arsenic-binding iron (oxy)hydroxide precipitates. These methods are widely recognized for their scalability in large-scale water treatment and their energy-efficient, low-temperature synthesis (<100 °C) [4,5]. (3) Enabling rapid adsorbent recovery via magnetic fields [14]. While prior work has focused on adsorption efficiency, few studies have systematically optimized desorption—a key factor for practical, cost-effective reuse [15].

This study bridges this gap by coupling magnetic adsorbent with statistical optimization for arsenate As(V) removal. Response Surface Methodology (RSM), a robust statistical tool for modeling multivariable systems [16,17,18], is employed to optimize desorption parameters (desorbent concentration, adsorbent dose, agitation speed) and to evaluate regenerability across sequential cycles. A three-factor, 2³ full factorial design with central composite design (CCD) is used to map interactions between variables, while kinetic models (Pseudo-First/Second-Order, Elovich) elucidate adsorption/desorption mechanisms.

Arsenic(V) was identified as the dominant species (95% vs. 5% As(III)) in contaminated water [19], underscoring its persistent threat to public health (the WHO safety limit is 10 µg/L). Though these concentrations appear low, chronic exposure risks long-term harm. To address this critical issue, our study investigates As(V) adsorption at 1 mg/L—a concentration mirroring real-world contamination—to advance safer water remediation strategies.

Arsenic contamination in groundwater, particularly as arsenate As(V), remains a critical global health challenge, with overexploited aquifers in regions like South Asia and South America disproportionately affected [20]. To address the limitations of conventional adsorbents—such as slow kinetics, poor selectivity, and difficulty in recovery—this study focuses on XMCs. These materials synergize hierarchical porosity (mesopores: 2–50 nm for enhanced As(V) diffusion; macropores: >50 nm for reduced mass transfer resistance) with uniformly dispersed Fe_3_O_4_ NPs (~5 wt.%, optimized to balance magnetic responsiveness and pore accessibility). This dual functionality enables not only high As(V) adsorption capacity (>95% uptake under optimized conditions).

By coupling advanced characterization (SEM/TEM for morphology, BET for surface area/pore distribution, FTIR for surface chemistry) with response surface methodology (RSM), we systematically optimize adsorption–desorption parameters (concentration, agitation, adsorbent dose) while elucidating the mechanistic roles of electrostatic interaction, ligand exchange, and pore filling. Furthermore, the study evaluates the material’s reusability across multiple cycles, addressing a critical gap in prior work on XMCs-based adsorbents. The integration of scalable synthesis (sol–gel pyrolysis), regeneration protocols, and magnetic recovery positions these functionalized xerogels as a sustainable, energy-efficient solution for decentralized water treatment systems.

## 2. Results and Discussion

### 2.1. Effect of Fe Content and Carbonization Temperature on As(V) Adsorption in Magnetic-Functionalized Carbon Xerogel

Gels were prepared via sol–gel polymerization using resorcinol (R, 1,3-dihydroxybenzene, C_6_H_4_(OH)_2_, 99.21%, Meyer), formaldehyde (F, HCHO, 37% methanol-stabilized solution, JT Baker), deionized water (W), and Fe_3_O_4_ NPs (M). Sodium carbonate (C, Na_2_CO_3_, anhydrous granules, JT Baker) was employed as the catalyst. Deionized water was used for all synthesis steps and solution preparations.

In this study, XMCs were synthesized with fixed molar ratios of R/C = 100, R/W = 0.04, and R/F = 0.5. The M/R ratio varied from 0.01 to 0.07, producing samples XMC7-850, XMC8-850, XMC9-850, and XMC10-850. The effect of iron (Fe) content and carbonization temperature on As(V) adsorption was evaluated under fixed conditions (pH 3, dose 2 g/L, As(V) concentration 1.024 mg/L, 24 h, 25 °C).

**Figure 1:**
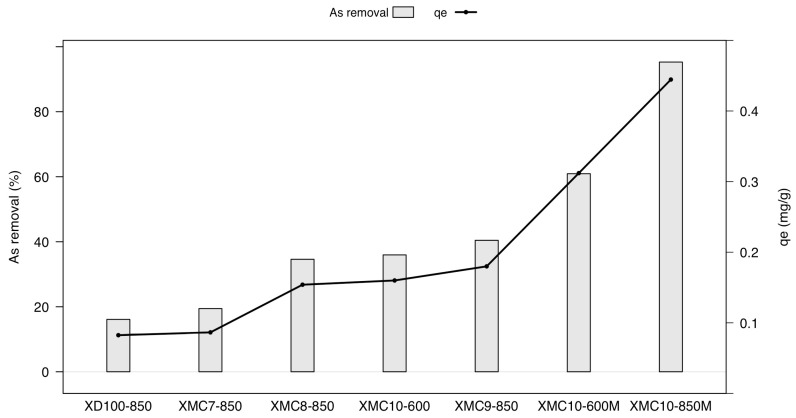
Effect of Fe contents and carbonization temperature in magnetic-functionalized carbon xerogel on the absorption of As(V) (Conditions: pH = 3, dose 2 g/L, As concentration 1.024 mg/L, 24 h and temperature 25 °C).

Figure 1 illustrates the arsenate adsorption performance of XMCs with varying M/R ratios and carbonization temperatures. Samples with lower M/R ratios—XMC7-850 (0.03), XMC8-850 (0.04), and XMC9-850 (0.05)—exhibited progressively lower As(V) removal efficiencies compared to XMC10-850 (M/R = 0.07). Increasing the M/R ratio from 0.01 to 0.07 enhanced removal efficiency and adsorption capacity by approximately 20%. Similar result found iron-modified composite exhibits high As(V) removal efficiency through surface complexation, enhanced by increased surface area and porosity [21].

XMC10-850M (carbonized at 850 °C) achieved the highest arsenate removal efficiency (95.28%) and adsorption capacity (0.445 mg/g), representing an 80% improvement over the non-magnetic xerogel (XD100-850). XMC10-600M (carbonized at 600 °C) demonstrated reduced performance compared to XMC10-850M, confirming that higher carbonization temperatures (850 °C) enhance pore development and stabilize Fe nanoparticles, thereby boosting adsorption. Surface modification with H_2_O_2_ further improved performance: XMC10-600M exhibited a 25% higher removal efficiency than XMC10-600 (carbonized at 600 °C without H_2_O_2_). These results highlight the synergistic effects of elevated carbonization temperatures (850 °C) and surface oxidation in optimizing As(V) adsorption.

The enhanced performance of H_2_O_2_-modified XMCs is attributed to the introduction of oxygen-containing functional groups (–OH, –COOH, –C–O, and –C=O) on the carbon surface. These groups, confirmed by increased transmittance values in spectroscopic analyses [22], improve As(V) affinity through electrostatic interactions and ligand exchange. Additionally, the surface charge of carbon materials plays a critical role in As(V) uptake [23], with oxidation increasing the density of negatively charged sites favorable for As adsorption [24].

Thus, optimizing Fe content (via M/R ratio), carbonization temperature, and surface functionalization enables the design of high-efficiency adsorbents for As(V) removal. Given the superior performance of XMC10-850M over other materials, this study investigated its adsorption kinetics and desorption optimization to highlight its viability as a reusable, high-efficiency adsorbent for arsenic-contaminated water.

### 2.2. Characterization of Materials

**Figure 2:**
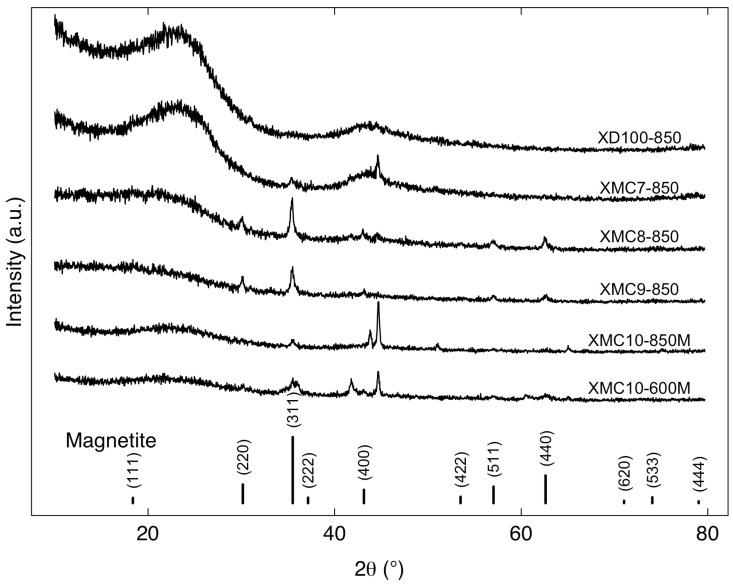
X-ray Diffraction (XRD) patterns of magnetic-functionalized carbon xerogels: effect of M/R ratios (0.01–0.07) and pyrolysis temperatures (600–850 °C).

Figure 2 presents the X-ray diffraction (XRD) spectra and diffraction patterns of magnetic-functionalized carbon xerogels, revealing both amorphous and crystalline phases. The amorphous component originates from the carbon xerogel matrix, while the crystalline phase corresponds to Fe_3_O_4_ NPs. The XRD patterns of the samples—including XD100-850, XMC7-850 (M/R = 0.03), XMC8-850 (M/R = 0.04), XMC9-850 (M/R = 0.05), XMC10-850M (M/R = 0.07), and XMC10-600M (M/R = 0.07)—revealed a broad peak centered between 2θ = 20–30°, characteristic of amorphous carbon. Distinct peaks at approximately 35.50°, 43.12°, 57.03°, and 62.63° were observed for samples XMC7-850, XMC8-850, XMC9-850, XMC10-850M, and XMC10-600M, corresponding to crystalline Fe_3_O_4_ (magnetite) nanoparticles, confirming successful incorporation into the carbon xerogel matrix [25].

The analysis demonstrates that samples with higher M/R ratios exhibit more intense crystalline peaks, confirming increased magnetite content. This trend is particularly evident when comparing XMC10-850M (M/R = 0.07) with lower-ratio samples. In contrast, the amorphous carbon xerogel matrix produces broad diffraction humps, characteristic of the disordered resorcinol-formaldehyde (RF) polymer structure [26]. Control samples XD100-850 (no NPs) and XMC7-850 (M/R = 0.01) show nearly identical patterns dominated by these amorphous features, with minimal crystalline peaks.

Comparing XMC10-600M and XMC10-850M, increasing the carbonization temperature from 600 °C to 850 °C resulted in slightly sharper magnetite peaks, reflecting enhanced Fe_3_O_4_ crystallinity at elevated temperatures. The effects of H_2_O_2_ surface modification, a strong oxidizing agent, were also considered. Hydrogen peroxide may oxidize Fe^2+^ ions in magnetite (Fe^2+^Fe^3+^_2_O_4_) to Fe^3+^, potentially inducing phase transformations in surface or near-surface regions [27]. This oxidation process could partially or fully convert Fe_3_O_4_ NPs to other phases, particularly at the surface.

**Figure 3:**
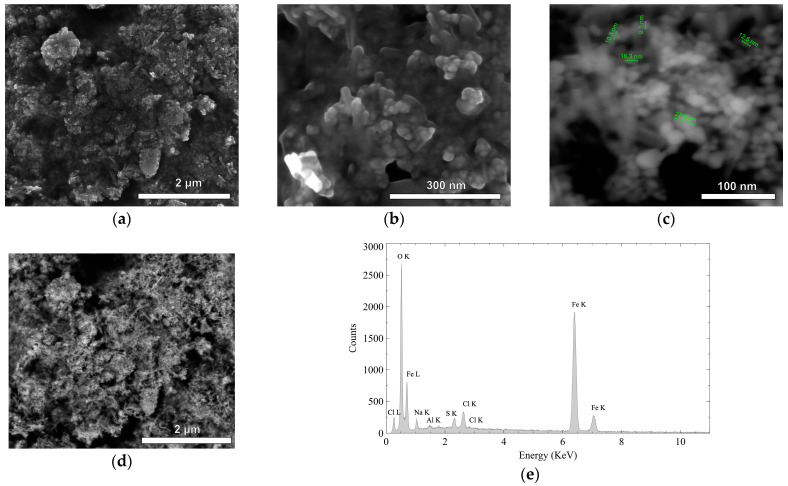
(**a**,**b**) Scanning electron microscopy (SEM) images at different magnifications, (**c**,**d**) backscattered electrons (BSE), and (**e**) Energy Dispersive X-Ray Spectroscopy (EDX) spectra of magnetite nanoparticles synthesized via conventional co-precipitation.

Scanning electron microscopy (SEM) images were obtained using the detection of secondary electrons (SE) and backscattered electrons (BSE) [28]. SEM images reveal the morphology of Fe_3_O_4_ NPs synthesized via conventional co-precipitation. At lower magnification (2500×, Figure 3a), the material exhibits aggregated particles forming a porous network [29]. Higher magnification (200,000×, Figure 3b) resolves individual nanoparticles with an estimated size range of 10–30 nm (Figure 3c), consistent with prior studies reporting Fe_3_O_4_ NPs of 16–33 nm diameter [30,31].

Backscattered electron (BSE) images (Figure 3d), which are sensitive to atomic number and material density, reveal higher atomic number elements (e.g., iron (Fe) and oxygen (O)) as brighter regions due to their stronger backscattering signal [28]. This indicates a homogeneous distribution of Fe_3_O_4_ NPs.

Energy-dispersive X-ray spectroscopy (EDX) spectra (Figure 3e) confirm the elemental composition, dominated by Fe and O, supporting successful iron oxide formation. Quantitative analysis (Figure 3e) reveals the following weight percentages: Fe (62.76%), O (23.0%), C (6.51%), Na (4.96%), Cl (2.03%), and S (0.73%). The high Fe and O content aligns with Fe_3_O_4_ stoichiometry [32].

Therefore, SEM-EDX validates the synthesis of Fe_3_O_4_ NPs (10–30 nm) with properties suitable for integration into carbon xerogels. While the nanoparticles are predominantly well-dispersed, localized agglomeration (Figure 3d) is attributed to magnetic interactions during synthesis, a phenomenon documented in similar systems [33]. These findings underscore the material’s potential for arsenic adsorption, balancing high surface reactivity with recoverability.

**Figure 4:**
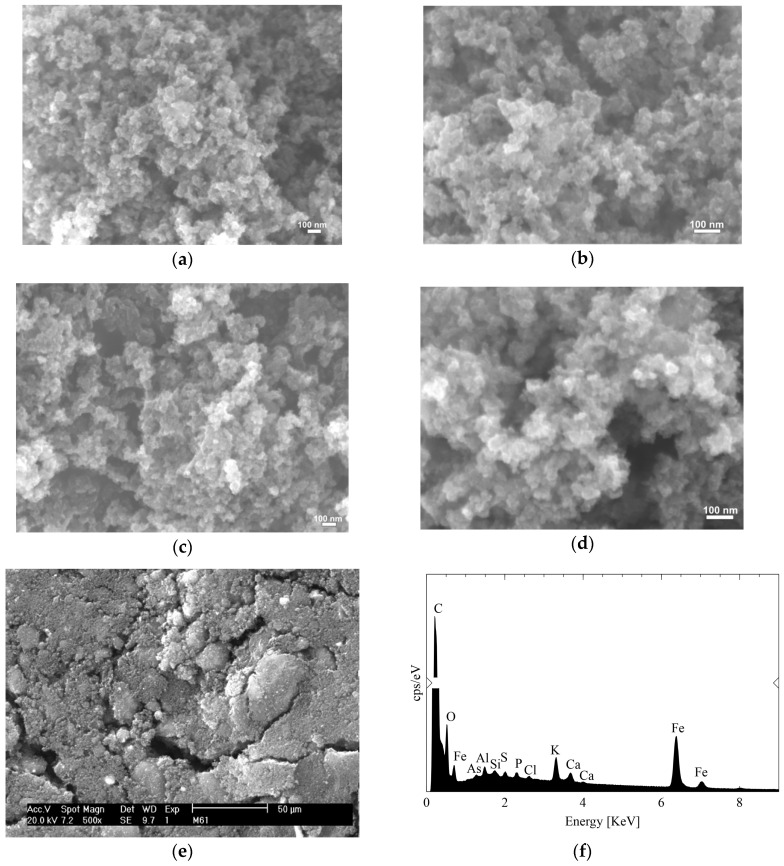
Scanning electron microscopy (SEM) images of magnetic-functionalized carbon xerogels: (**a**,**b**) XMC10-600M; (**c**,**d**) XMC10-850M before As(V) adsorption; (**e**) XMC10-850M after As(V) adsorption; and (**f**) energy-dispersive X-ray spectroscopy (EDS) spectrum of XMC10-850M [35].

Figure 4 illustrates the surface morphology and porous structure of XMCs, characterized using scanning electron microscopy (SEM) at magnifications of 50,000× (Figure 4a,c) and 100,000× (Figure 4b,d). XMC10-600M and XMC10-850M were synthesized with fixed ratios (R/W = 0.04, R/C = 100, M/R = 0.07) and carbonized at 600 °C and 850 °C, respectively. All images (Figure 4a–d) reveal a highly porous and interconnected structure characteristic of xerogels. Comparing the two materials, the SEM analysis reveals that both XMC10-600M and XMC10-850M consist of agglomerated nanoparticles. However, the higher carbonization temperature used for XMC10-850M resulted in a noticeably more open and porous texture within the agglomerates compared to the denser structure of XMC10-600M. This enhanced porosity and likely higher accessible surface area in XMC10-850M facilitate faster diffusion of arsenic to the binding sites and provide a greater number of active sites [34].

Figure 4d,e presents SEM images and EDX analysis of XMC10-850M after As(V) adsorption, respectively, revealing key morphological and compositional characteristics. The SEM micrograph at 500× magnification shows a heterogeneous surface with aggregated particles, maintaining the typical structure of magnetic carbon xerogels. EDX spectra confirm the presence of C and O from the xerogel matrix, Fe from Fe_3_O_4_ NPs, and critically, arsenic (As), providing direct evidence of successful contaminant uptake. The Fe content (3.56 wt%) determined by EDX aligns with atomic absorption spectroscopy results, validating successful magnetite incorporation. The relative intensity of the arsenic peak (0.3 wt%) offers semi-quantitative insight into adsorption efficiency, while the preserved iron signal indicates maintained magnetic functionality. These results collectively demonstrate that XMC10-850M retains its structural integrity after As(V) adsorption while effectively capturing the target contaminant, confirming its dual capability as both an efficient adsorbent and magnetically recoverable material for water treatment applications [4]. The complementary SEM-EDX analysis provides comprehensive verification of the material’s performance, linking its morphological features to its adsorption capacity and recovery potential.

**Figure 5:**
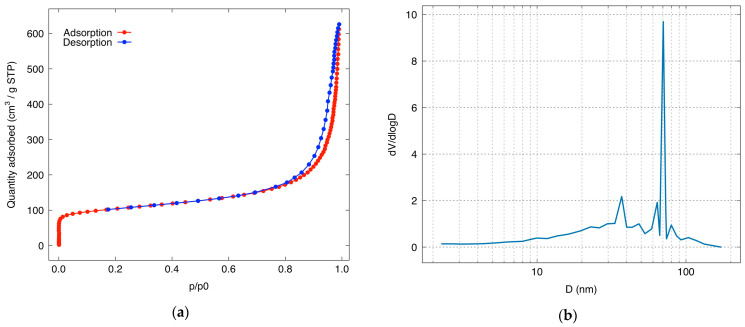
(**a**) Nitrogen adsorption–desorption isotherm and (**b**) pore size distribution of XMC10-850.

The textural properties of the XMC10-850M were analyzed via nitrogen adsorption–desorption isotherms (Figure 5a), which exhibited a Type IV profile characteristic of mesoporous materials [36]. A distinct hysteresis loop confirmed capillary condensation within the pore network. The Brunauer–Emmett–Teller (BET) surface area of XMC10-850M was 378.8 m^2^/g, lower than the RFX blank (399.19 m^2^/g), likely due to Fe_3_O_4_ NPs occupying pore volume. Pore size distribution, determined using Barrett–Joyner–Halenda (BJH) (Figure 5b), revealed a non-uniform mesoporous structure (2–50 nm), contrasting with the RFX blank’s uniform mesopores (average diameter: 5.23 nm). This structural divergence highlights the impact of magnetite incorporation on pore architecture.

BJH analysis reveals the pore structure of the XMC10-850M material, specifically its adsorption pore distribution, as presented in Table 1. The cumulative pore volume measured via BJH adsorption is 0.9035 cm^3^/g, indicating a high capacity for adsorbing molecules within the pore network. The cumulative pore volume (0.9036 cm³/g) was dominated by macropores (>50 nm, 54.07%), with a median pore width of 18.07 nm—significantly larger than RFX’s 5.23 nm. Bimodal peaks at 2.16 nm (small mesopores) and 59.82 nm (larger pores) indicated a broad pore size distribution. The pore volume distribution showed contributions from smaller mesopores (2–10 nm: 15.22%, 0.1375 cm³/g), medium mesopores (10–20 nm: 10.26%, 0.0927 cm³/g), and larger mesopores (20–50 nm: 20.45%, 0.1848 cm³/g). This heterogeneity suggests that magnetite integration disrupts the carbon matrix, introducing macroporosity while retaining critical mesopore volume for adsorption. Compared to RFX’s uniform mesoporous network, XMC10-850M’s complex pore architecture—marked by wider pore distribution and macroporosity—likely enhances mass transfer kinetics, compensating for its reduced surface area.

**Figure 6:**
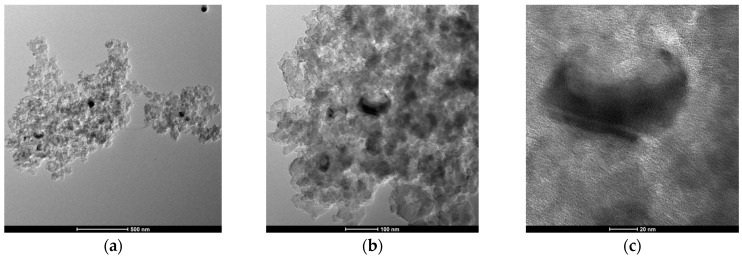
(**a**–**c**) Transmission electron microscopy (TEM) images with different magnifications of magnetic-functionalized carbon xerogels (XMC10-850M) at increasing magnifications.

Transmission electron microscopy (TEM) was employed to characterize the microstructure of the XMC10-850M composite. The TEM images at progressive magnifications (Figure 6a–c) reveal the material’s hierarchical organization. At lower magnification (Figure 6a), the micrograph displays a continuous, interconnected carbonaceous network. Higher magnifications (Figure 6b) clearly demonstrate the homogeneous dispersion of Fe_3_O_4_ NPs within the carbon matrix. These electron-dense nanoparticles, visible as dark contrast regions, exhibit sizes ranging from 5 to 15 nm with uniform distribution.

Figure 6c reveals crystalline lattice fringes at the Fe_3_O_4_–carbon interface, confirming both the nanoparticles’ crystallinity and their intimate contact with the carbon support. This nanostructural arrangement features well-dispersed, nanometer-sized Fe_3_O_4_ particles within a porous carbon framework to provide an optimal architecture for As(V) adsorption, offering abundant surface active sites and facilitating efficient contaminant capture.

**Figure 7:**
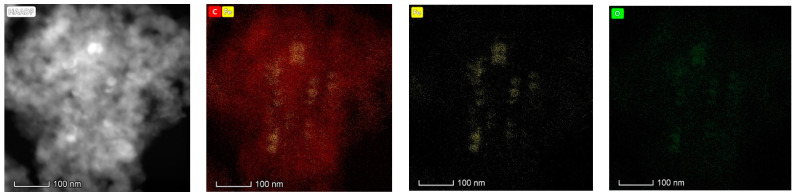
HAADF-STEM Imaging and Elemental Mapping of XMC10-850M Composite.

The high-angle annular dark-field (HAADF) image in Figure 7 reveals the nanostructural organization of the XMC10-850M composite, with brighter regions corresponding to iron oxide nanoparticles (due to their higher atomic number) embedded within a darker carbon xerogel matrix. Elemental mapping confirms the homogeneous distribution of constituent elements (C, Fe, O, Cl), consistent with SEM-EDS results, while energy-dispersive spectroscopy (EDS) validates the material’s composition. Notably, Fe (depicted as yellow spots in the elemental map) exhibits uniform dispersion at a low concentration of ~5 wt% within the RF-derived carbon xerogel. Complementary HAADF imaging and elemental mapping (Figure 7) further clarify the spatial distribution: carbon (red) forms a continuous framework, while iron (yellow) localizes as well-dispersed Fe_3_O_4_ NPs clusters. This nanostructural architecture—crystalline Fe_3_O_4_ NPs seamlessly integrated into an amorphous carbon xerogel—confirms successful nanocomposite synthesis. The intimate Fe_3_O_4_–carbon interface not only enhances As(V) adsorption capacity through increased active sites but also facilitates efficient magnetic separation, underscoring the material’s dual functionality.

**Figure 8:**
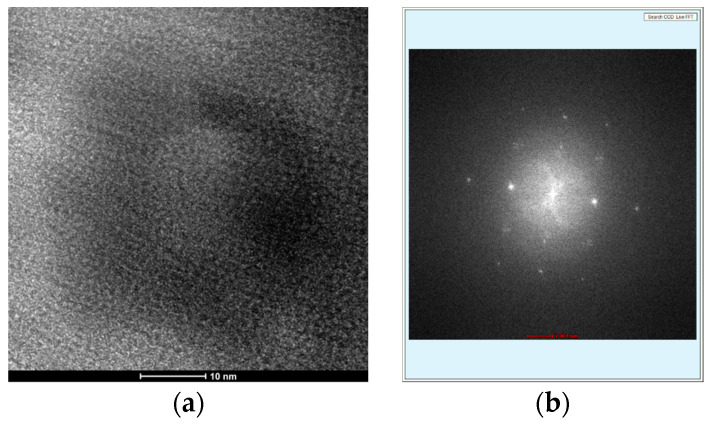
(**a**) HRTEM micrograph showing Fe_3_O_4_ NPs (dark contrast) embedded in the carbon xerogel matrix of XMC10-850M. (**b**) SAED pattern confirming magnetite crystallinity (bright spots) and amorphous carbon (diffuse rings).

Figure 8 presents a high-resolution TEM (HRTEM) micrograph and the corresponding Selected Area Electron Diffraction (SAED) pattern of the magnetic carbon xerogel XMC10-850M. The HRTEM image reveals a nanostructure composed of crystalline Fe_3_O_4_ NPs embedded within an amorphous carbon matrix. Distinct lattice fringes in the crystalline regions confirm the atomic ordering of the magnetite phase, while the surrounding amorphous matrix is characteristic of the RF-derived carbon xerogel. Bonding rearrangements between the Fe_3_O_4_ NPs and the carbon matrix, consistent with prior TEM studies [37], suggest strong interfacial interactions. Complementing these observations, the SAED pattern demonstrates the material’s dual amorphous–crystalline nature: diffuse rings correspond to the amorphous carbon framework, while bright diffraction spots match the crystalline planes of Fe_3_O_4_, confirming magnetite’s crystallinity. The entrapped Fe_3_O_4_ NPs, sized between 10 and 20 nm, align with HRTEM observations and further corroborate the composite’s structural integrity. Together, these analyses confirm XMC10-850M’s architecture—well-dispersed crystalline Fe_3_O_4_ NPs within a continuous amorphous carbon xerogel matrix—optimized to enhance interfacial interactions critical for adsorption and magnetic separation performance.

**Figure 9:**
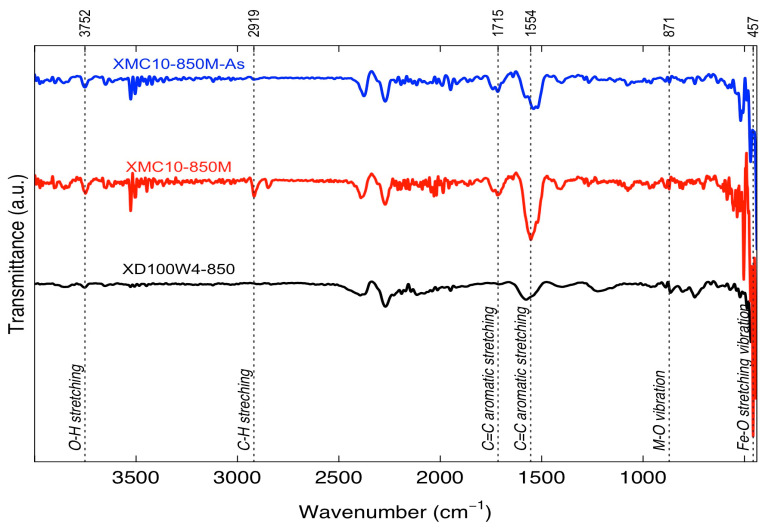
FTIR Spectra of magnetic-functionalized carbon xerogels XMC10-850M before and after As(V) adsorption and carbon xerogel XD100-850.

FTIR analysis was used to identify functional groups in magnetic-functionalized carbon xerogels before and after As(V) adsorption. The FTIR spectra of the XMC samples are presented in Figure 9. Prior to As(V) adsorption, the XMC10-850M spectrum exhibits bands similar to those of the pristine carbon xerogel, including a broad -OH stretching band (~3400 cm^−1^) and a C=C aromatic stretching band (~1600 cm^−1^). A distinct band at ~580 cm^−1^, characteristic of Fe–O stretching vibrations in Fe_3_O_4_ NPs, confirms the successful incorporation of Fe_3_O_4_ NPs into the XMC10-850M composite.

After pyrolysis, several IR bands disappeared, particularly the -OH group signal at ~3500 cm^−1^. These findings align with studies by Sun T. et al. (2018) [38], who reported that oxygen- or hydrogen-containing functional groups in RF polymers pyrolyzed at 800 °C are undetectable due to the dominance of carbonaceous material.

However, residual adsorption bands were observed at 2923 cm^−1^ (C-H stretching), 1716 cm^−1^ and 1543 cm^−1^ (C=C aromatic stretching), and 1444 cm^−1^ (C-H bending vibration), attributed to unpyrolyzed resorcinol molecules. These spectral peaks match those reported in similar studies [39,40]. Additionally, bands at 1444 cm^−1^ (Fe=O stretching), 1090 cm^−1^ (M–OH stretching), and 501 cm^−1^ (Fe–O stretching) for XMC10-850M confirm the presence of magnetite in the RF gel composite [41,42].

Following As(V) adsorption, the FTIR spectrum of XMC10-850M reveals significant alterations compared to its pre-adsorption state. The reduced intensity of the -OH band at ~3400 cm^−1^ suggests hydroxyl group participation in arsenic binding. Similarly, diminished intensities of the 2923 cm^−1^ (C–H stretching) and 1543 cm^−1^ (C=C aromatic stretching) bands—corresponding to aromatic C–C bonds in the resorcinol–formaldehyde matrix—indicate arsenate interaction with the carbon framework [40,42]. Additional spectral shifts in C–O and Fe–O stretching bands further imply arsenic coordination with surface functional groups. Notably, reduced intensities at 1444 cm^−1^ (Fe=O) and 501 cm^−1^ (Fe–O) [42], along with altered band profiles, confirm magnetite’s role in As(V) adsorption.

Therefore, the FTIR analysis suggests that As(V) adsorption onto XMC10-850M involves interactions with both hydroxyl groups on the carbon surface and Fe-O groups on the Fe_3_O_4_ NPs. The changes observed in the FTIR spectra after As(V) adsorption support the hypothesis that surface complexation and ligand exchange mechanisms play a role in As(V) removal by these magnetic-functionalized carbon xerogels [43]. The adsorption mechanism of arsenate on magnetite-functionalized carbon xerogels likely involves both physical and chemical processes. Fe_3_O_4_ NPs exhibit a positively charged surface due to Fe^2+^ ions, facilitating electrostatic attraction of negatively charged arsenic species (HAsO_4_^2^−, H_2_AsO_4_−, H_3_AsO_4_) in solution [44].

The point of zero charge (pHpzc) values for XMC10 and XMC10-850M were 4.77 and 8.03, respectively. The pHpzc represents the pH at which a material’s surface charge is neutral. Carbonizing organic xerogels via thermal treatment increases their pHpzc, as carbonization removes surface functional groups (e.g., hydroxyl [-OH], carboxyl [-COOH], and phenolic groups), resulting in materials with high carbon content and thermally stable nanostructures [45]. This process reduces surface acidity/basicity and enhances structural homogeneity.

Surface chemistry in activated carbons is governed by oxygen-containing complexes, which determine charge distribution through the dissociation of acidic or basic groups [36]. These complexes also influence surface hydrophobicity. During carbonization, graphitic structure formation and reduced surface heterogeneity drive the pHpzc increase. As(V) adsorption on magnetic-functionalized carbon xerogels is mediated by Coulombic attraction: the positively charged XMC surfaces electrostatically attract negatively charged arsenic ions (HAsO_4_^2^−, H_2_AsO_4_−), enabling adhesion and removal from solution [42]. Reduced functional group density decreases absorption band intensity, as fewer molecules are available to interact with radiation. Functional groups may form bonds (e.g., chemical or hydrogen bonding) or interact via electrostatic forces [42]. Thus, As(V) removal likely involves chemical adsorption via bond formation between ions and surface functional groups on the carbon xerogel surface.

### 2.3. Kinetic As(V) Adsorption Analysis

**Figure 10:**
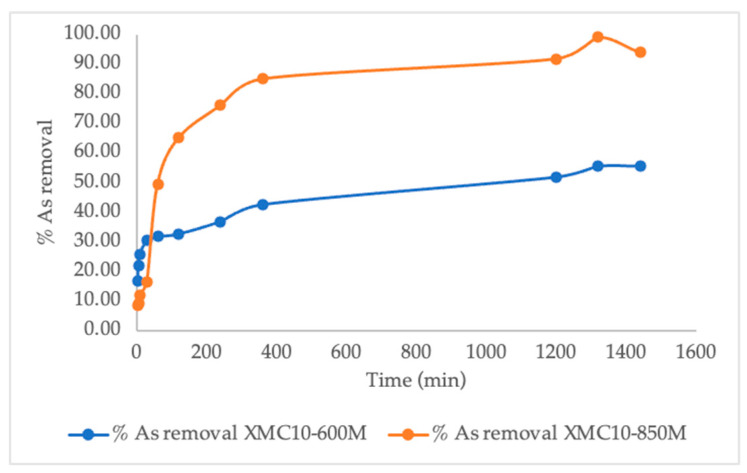
Effect of time of Arsenic adsorption using XMC10-600M and XMC10-850M prepared at carbonization temperatures of 600 °C and 850 °C, respectively (conditions: adsorbent dosage = 2 g/L, pH = 3.0, initial arsenic concentration = 1.024 mg/L).

Figure 10 compares the effect of time of arsenic adsorption of XMC10-600M and XMC10-850M (carbonized at 600 °C vs. 850 °C) under acidic conditions (pH 3.0, 2 g/L dose, 1.024 mg/L As), revealing that XMC10-850M achieves superior performance: ~85% removal in 6.7 h and ~99% equilibrium capacity, far exceeding XMC10-600M’s 55% removal after 23 h. This enhancement is attributed to higher carbonization temperature optimizing porosity, surface area, crystallinity (e.g., magnetite/SiO_2_ phases), and surface chemistry, which favor arsenic binding. The rapid initial adsorption (both materials) reflects abundant active sites, while slower equilibrium phases highlight site saturation [46]. Practically, XMC10-850M’s near-complete removal within ~7 h underscores its viability for acidic wastewater treatment, aligning with dominant arsenic species (H_3_AsO_4_/H_3_AsO_3_) and adsorbent surface charge. These findings emphasize thermal treatment’s critical role in designing efficient adsorbents.

Table 2 presents a detailed analysis of the As(V) adsorption kinetics onto XMC10-600M and XMC10-850M, using four kinetic models: Pseudo-First-Order (PFO), Pseudo-Second-Order (PSO), Elovich, and Power. The table includes the model parameters (q_e_, k_1_, k_2_, β, α, a, b), the coefficient of determination (R^2^) and Root Mean Squared Error (RMSE).

For all models, the RMSE values are consistently lower for XMC10-850M compared to XMC10-600M, reinforcing that the higher carbonization temperature improves model fit and better represents the kinetic process. Under experimental conditions (pH 3, 2 g/L dose, 1.024 mg/L As concentration, 24 h contact time, 26 ± 1 °C), the adsorption capacities (q_t_) of XMC10-600M and XMC10-850M were 0.22 mg/g and 0.498 mg/g, respectively. The Elovich model provides a strong fit for XMC10-850M, evidenced by its high R^2^ (0.9396) and low RMSE (40.89 µg/g). However, the Power model outperforms other models for both XMC10-600M and XMC10-850M, achieving the highest R^2^ (0.9443) and lowest RMSE (39.30 µg/g), particularly for XMC10-850M. Based on R^2^ optimization and minimized RMSE values, the Elovich and Power models best describe As(V) adsorption kinetics for XMC10-850M, suggesting a process governed by surface heterogeneity and potential diffusion limitations [46]. All kinetic models were applied to both materials; however, XMC10-850M (850 °C) demonstrated superior and more reliable kinetic data with better model fit compared to XMC10-600M (600 °C).

### 2.4. Study of Acidic and Alkaline Regeneration Agents for Arsenic Desorption from Magnetic-Functionalized Carbon Xerogels

The results revealed significant variation in desorption efficiency across the tested desorbing agents. Nitric acid (HNO_3_, 0.10 M) demonstrated the highest arsenic recovery (40.18%), attributed to its strong oxidative and acidic properties, which likely disrupted arsenic–surface interactions by protonating adsorption sites or dissolving iron oxides within the xerogel matrix [47,48]. Hydrochloric acid (HCl, 0.10 M) showed moderate efficiency (22.32%), possibly due to Cl− ion exchange or partial surface protonation [48]. Potassium hydroxide (KOH, 0.10 M) achieved limited desorption (17.86%), suggesting weak competition between OH− ions and arsenate anions for binding sites [35]. Notably, sodium hydroxide (NaOH) and acetic acid (CH_3_COOH) exhibited no detectable arsenic release, likely due to re-precipitation of arsenic at high pH (NaOH) or insufficient acidity (CH_3_COOH) to destabilize arsenic complexes.

The superior performance of HNO_3_ highlights its potential for arsenic recovery, though its corrosiveness and environmental toxicity raise practical concerns. Conversely, the inefficacy of NaOH and CH_3_COOH underscores their unsuitability under these conditions. Material stability tests indicated that the magnetic-functionalized carbon xerogels retained structural integrity during acidic treatment, though prolonged exposure to HNO_3_ may degrade organic components. For practical applications, future studies should optimize HNO_3_ and KOH concentration, assess adsorbent reusability across multiple cycles, and explore eco-friendly alternatives.

### 2.5. Optimizing Arsenic Desorption Using Response Surface Methodology

#### 2.5.1. RSM Analysis for Arsenic Desorption Using XMC10-850M and HNO_3_

The RSM study for arsenic desorption using HNO_3_ highlights HNO_3_ concentration as the most critical parameter, with efficiency rising sharply above 0.15 mg/L due to enhanced ionic competition displacing arsenate from adsorption sites.

**Figure 11:**
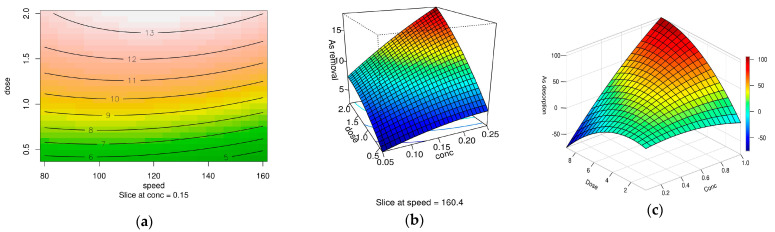
(**a**) Contour plot (dose vs. speed at fixed HNO_3_ concentration = 0.15 mg/L). (**b**) 3D surface plot (dose vs. HNO_3_ concentration at fixed speed = 160.4 rpm), and (**c**) prediction 3D surface plot (dose vs. HNO_3_ concentration).

The contour plot analysis (Figure 11a) at a fixed HNO_3_ concentration (0.15 mg/L) reveals minimal sensitivity to adsorbent dose (≤1.2 g/L) and agitation speed (120–160 rpm), confirming the inadequacy of low HNO_3_ levels for effective desorption. In contrast, the 3D surface plot (Figure 11b) demonstrates a parabolic relationship between HNO_3_ concentration and adsorbent dose at a fixed speed (160.4 rpm), peaking at 0.25 mg/L HNO_3_ and 2.0 g/L dose. This synergy reflects optimized ionic competition (via elevated HNO_3_) and efficient adsorbent contact (via moderate dosing), balancing efficacy and cost. Adsorbent dose exhibits a threshold effect (plateauing beyond 1.2 g/L), while agitation speed primarily ensures mixing rather than kinetic enhancement.

Figure 11c illustrates the relationship between HNO_3_ concentration (Conc) and As(V) desorption efficiency from the magnetic-functionalized carbon xerogel XMC10-850M, based on an RSM study. Arsenic desorption efficiency increases significantly with higher HNO_3_ concentrations, peaking at 1.0 M at dose = 8.5 g/L. Lower concentrations (0.2–0.6 M) show progressively reduced performance, highlighting the critical role of acid strength in mobilizing arsenate ions through enhanced ionic competition or pH-driven mechanisms. The optimal condition shows in Figure 11c, the highest desorption efficiency occurs at 1.0 M HNO_3_ at 8.5 g/L, suggesting this concentration maximizes arsenate recovery by overcoming adsorption site retention.

The model’s robustness is validated by a high R^2^ (>0.90) and non-significant lack of fit (*p* > 0.05), confirming its reliability for predicting arsenic recovery. Optimal conditions (≥0.25 mg/L HNO_3_, 1.2–2.0 g/L dose, 120–160 rpm) prioritize cost-effectiveness without compromising efficiency. These findings underscore the necessity of parameter optimization, particularly HNO_3_ concentration and dose, to design scalable, sustainable water treatment systems. Future work should explore higher HNO_3_ concentrations and validate the model in real groundwater matrices to address complex environmental challenges.

#### 2.5.2. RSM Analysis for As(V) Desorption Using XMC10-850M and KOH

RSM analysis, employing a central composite design, evaluates the desorption efficiency of arsenic from XMC10-850M using KOH as the regenerant. While the study identifies optimal conditions (1.0 M KOH, 150 rpm agitation, 180 min contact time), the second-order model’s 3D surface and contour plots inadequately visualize the relationships between the factors—KOH concentration (*x*_1_), agitation speed (*x*_2_), and dose (*x*_3_)—and their combined impact on desorption. Despite this limitation, the regression model demonstrates strong statistical validity, with an R^2^ of 0.92 and *p*-value < 0.05, confirming its ability to explain 92% of the variance in desorption efficiency. The non-significant lack of fit (*p* > 0.05) further validates the model’s reliability. The regression Equation 1 derived from the data is as follows:

As desorption = 49.0661 + 2.3998*x*_1_ + 3.7816*x*_2_ + 29.3671*x*_3_ − 2.5448*x*_1_*x*_2_ + 3.837*x*_1_*x*_3_ + 4.9842*x*_2_*x*_3_ − 8.8971*x*_1_^2^ + 11.0396*x*_2_^2^ − 3.1746*x*_3_^2^ (1)

with the coded variables *x*_1_ (conc), *x*_2_ (speed), and *x*_3_ (dose).

Therefore, the optimal desorption conditions (1.05 M KOH, 160 rpm agitation speed, and 2 g/L adsorbent dose) achieved 95.3% As(V) removal efficiency with a standard error (SE) of 9.76 and a 95% confidence interval (CI) of 71.5–119.2.

RSM analysis revealed that spent adsorbent dose (*x*_3_) is the most significant factor influencing As(V) desorption, with a coefficient of 29.37 and a statistically significant *p*-value (<0.001). This suggests that increasing the adsorbent dose enhances desorption efficiency, likely due to greater availability of active sites for interaction with KOH. Furthermore, a higher KOH concentration directly improves arsenic recovery, attributed to enhanced ionic competition (KOH displacing arsenate ions) and pH-driven mobilization. In contrast, KOH concentration (*x*_1_) and agitation speed (*x*_2_) showed no significant effects (*p* > 0.05), indicating minimal impact on desorption efficiency under the tested conditions.

The second-degree polynomial model obtained (R^2^ = 0.9237) explains 92.37% of the variability in the data, demonstrating high predictive capability. Although the adjusted R^2^ (0.8092) is lower, this is attributed to the inclusion of non-significant terms (e.g., interactions and quadratic terms), common in complex RSM models. The overall significance of the model is confirmed with a *p*-value = 0.0097 (<0.05), while the non-significance of the lack of fit (*p* = 0.606) validates the absence of systematic errors, ensuring that the model adequately fits the experimental data.

**Figure 12:**
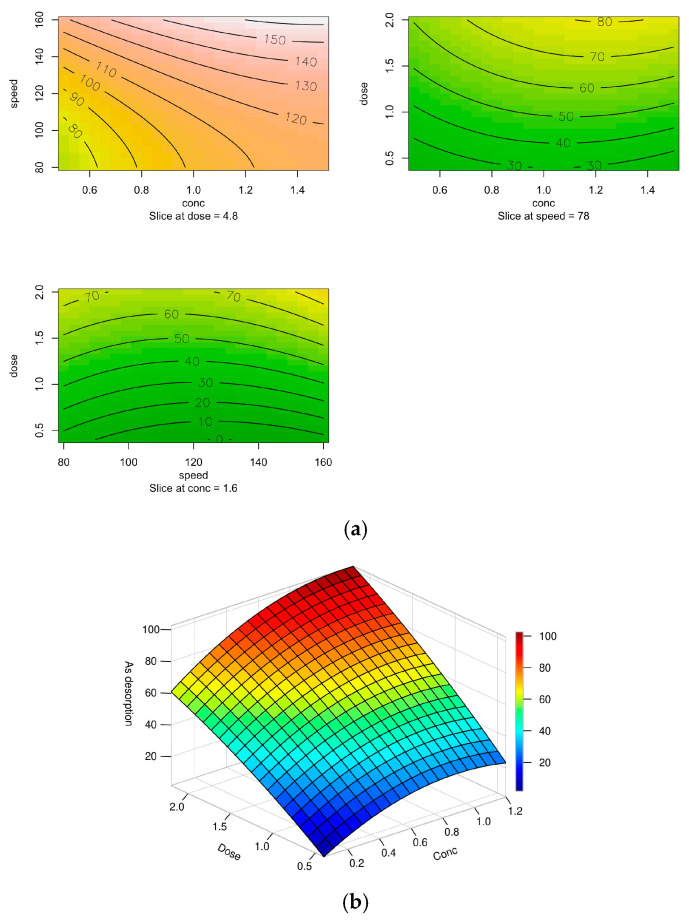
(**a**) 2D contour plot of As(V) desorption efficiency: effects of KOH concentration, agitation speed, and dose (RSM-optimized parameters) and (**b**) 3D surface plot of As(V) desorption: KOH concentration vs. dose at fixed speed (160 RPM) [35].

Figure 12 presents contour plots and a 3D surface plot depicting the desorption study of arsenic from As(V)-loaded XMC10-850M, based on a second-order model. The plots illustrate the relationship between different factors (concentration, speed, and dose) and arsenic desorption efficiency.

The contour plots (Figure 12a) display the relationship between speed and concentration, dose and concentration, and dose and speed of agitation with stationary points (optimized parameters: concentration = 1.64 M, speed = 77.79 RPM, dose = 4.85 g/L) derived via RSM. They indicate that arsenic desorption is favored at KOH concentrations between 1 and 1.4 M. Specifically, increasing KOH concentration alongside higher doses, while maintaining medium agitation speed, achieves maximum desorption rates. The 3D surface plot (Figure 12b) further illustrates the combined effect of KOH concentration and dose on arsenic desorption at a fixed agitation speed of 160 RPM with stationary points estimated via RSM. The highest desorption efficiencies (95.34%) occur when KOH concentration and dose range from 1.05 M to 2 g/L.

The findings of this study demonstrate that arsenic desorption from XMC10-850M can be effectively optimized using a second-order model integrated with RSM. The RSM framework successfully identified the optimal regeneration parameters: 1.0 M KOH, 2 g/L adsorbent dose, and 160 RPM agitation speed, achieving maximum desorption efficiency. These results align with prior studies that validate RSM’s utility in enhancing arsenic recovery from spent adsorbents, such as activated carbon, underscoring its robustness in parameter optimization for environmental remediation processes [48,49].

### 2.6. Kinetic Study of As(V) Desorption from XMC10-850M Carbon Xerogels

**Figure 13:**
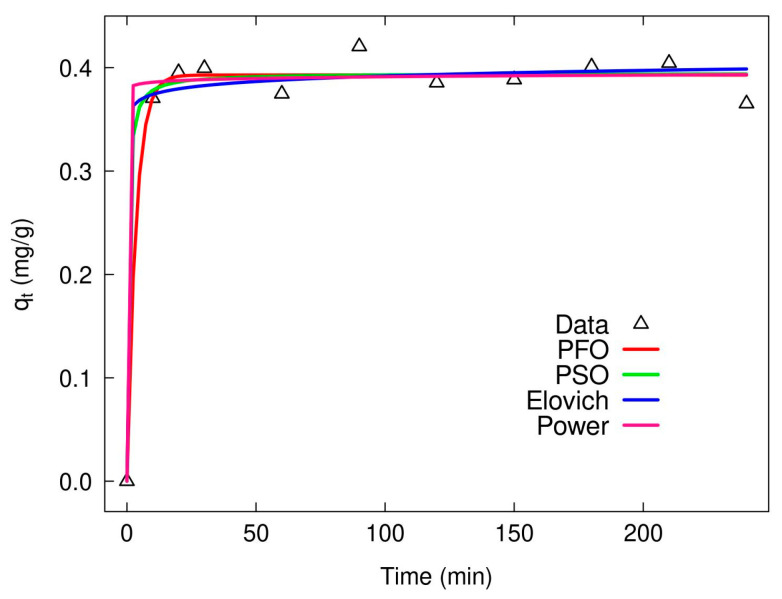
Kinetic study of As(V) desorption from XMC10-850M carbon xerogels.

The kinetic analysis of As(V) desorption from spent As(V)-loaded XMC10-850M carbon xerogels, evaluated using four models (Pseudo-First-Order, Pseudo-Second-Order, Elovich, and Power Equation; Figure 13), revealed rapid desorption dynamics that stabilize shortly after 15 min, with nearly complete arsenic recovery achieved within 90 min. All models demonstrated high effectiveness, showing nearly identical fits to experimental data, suggesting robust agreement in describing the desorption process despite differing mechanistic assumptions (e.g., surface heterogeneity versus diffusion control).

During the initial phase, the desorbed amount (q_t_) rose sharply from 0 to ~0.38 mg/g, indicating swift release of weakly bound adsorbates or molecules occupying readily accessible surface sites. This kinetic behavior aligns with physisorption-dominated processes, where van der Waals forces or weak interfacial interactions govern adsorption–desorption equilibria. The efficiency of this phase highlights the potential for rapid adsorbent regeneration—a critical advantage for cyclic reuse applications.

Approximately 78% of arsenic was released within the first 10 min, underscoring the rapid liberation of weakly bound surface species. Desorption efficiency peaked at 88.7% (q_t_ = 0.395 mg/g) by 90 min, suggesting partial equilibrium attainment, likely driven by the release of arsenate ions from accessible sites (e.g., outer pores or electrostatic interactions). Beyond 20 min, the marked decline in desorption rate signaled a transition to a diffusion-limited regime, where residual adsorbates in deeper pores or stronger binding sites require extended diffusion pathways or higher activation energies for release [50].

Non-monotonic post-peak behavior—evident in fluctuations such as a decline to 79.1% at 60 min and a final drop to 77.1% at 240 min—suggests potential re-adsorption phenomena, competition with counter-ions, or heterogeneous binding site interactions. The incomplete equilibrium after 240 min implies contributions from stronger chemisorption mechanisms (e.g., inner-sphere complexes with iron oxides in the magnetic xerogel matrix) or intra-particle diffusion limitations, where arsenic trapped in micropores necessitates prolonged release times.

The magnetic component (Fe_3_O_4_) likely stabilizes arsenic through Fe–O–As bonds, as indicated by FTIR data, which inhibits easy desorption under the tested conditions. These findings highlight the material’s dual adsorption–desorption behavior: rapid regeneration (~90 min) enables reuse, but persistent binding sites or structural irregularities restrict complete arsenic recovery.

### 2.7. As(V) Adsorbent Regeneration

**Figure 14:**
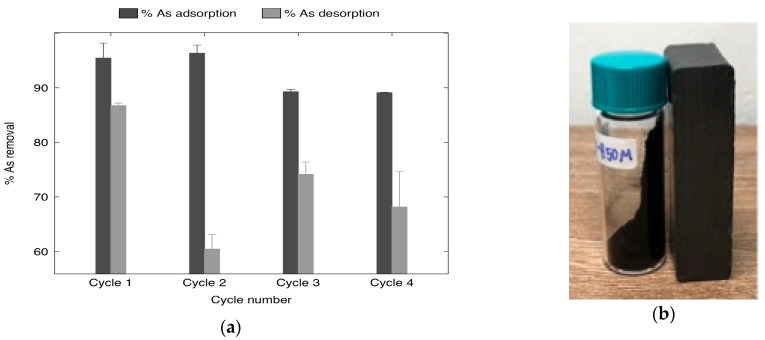
(**a**) As(V) adsorption–desorption efficiency of XMC10-850M over four consecutive cycles under RSM-optimized conditions (error bars = standard deviation) [35], and (**b**) magnetization behavior of magnetic-functionalized carbon xerogels (XMC10-850M).

The regeneration capability of XMC10-850M (Figure 14a) was evaluated under optimized conditions (1.0 M KOH, 160 rpm, 90 min), demonstrating high initial adsorption efficiency (~95.42% As(V) removal in Cycle 1) and sustained performance (>89% across all four cycles) with minimal variability of standard deviation. The material exhibits reliable and consistent arsenic adsorption across four cycles, indicated by low and decreasing standard deviations.

The desorption data, while preliminary, show that desorption efficiency exhibited significant instability, declining sharply from ~86.72% in Cycle 1 to ~60.44% in Cycle 2 before stabilizing at ~68.17–74.12% in subsequent cycles. It suggests increasing variability in regeneration efficiency over four cycles (C1–C4), as evidenced by rising standard deviations (Figure 14a). This inconsistency suggests progressive retention of As(V) in stronger binding sites or deeper pores, where diffusion limitations hinder complete elution [50]. However, the desorption process becomes increasingly unreliable and variable with repeated use, as shown by the rising standard deviations.

A critical challenge observed was the escalating material loss (~19.9–39.7% cumulative loss per cycle). The fine particulate nature of XMC10-850M renders it prone to mechanical entrapment or leakage during washing process. This operational limitation artificially inflates material loss, masking the adsorbent’s inherent stability. Future studies should explore pelletizing XMC10-850M to minimize handling losses, enhance mechanical integrity, and improve compatibility with industrial-scale filtration systems.

The magnetic-functionalized carbon xerogels demonstrate magnetic permeability behavior [4] due to being strongly attracted to magnets across multiple regeneration cycles, resulting in significant magnetic susceptibility as shown in Figure 14b. This characteristic allows for their facile magnetic isolation and retrieval from aqueous environments using an external magnetic field [31]. Furthermore, they display favorable solid–liquid separation properties in aqueous matrices.

## 3. Conclusions

This study demonstrates the breakthrough potential of magnetic-functionalized carbon xerogels (XMC10-850M) as a sustainable, high-capacity adsorbent for arsenic removal, synthesized via ultrasound-assisted integration of Fe_3_O_4_ NPs (15–20 nm) into a resorcinol–formaldehyde matrix. Characterization revealed a hierarchical porous structure (BET surface area: ~378.8 m^2^/g) with uniform Fe_3_O_4_ dispersion and H_2_O_2_-induced surface modifications that amplified arsenate affinity through enhanced active site accessibility. Adsorption kinetics aligned with the Elovich and Power Function models, underscoring heterogeneous chemisorption and pore diffusion as rate-limiting steps. Optimized regeneration via Response Surface Methodology (RSM) achieved >88% efficiency retention over four cycles using 1.0 M KOH, with rapid 90 min desorption kinetics, highlighting practical scalability for water treatment systems. While XMC10-850M excels in adsorption capacity (∼0.5 mg/g) and magnetic recovery, operational losses during washing and incomplete desorption (∼12% capacity decline per cycle) reveal a critical trade-off between performance and regenerability. Future research should prioritize material form optimization (e.g., pelletization) to minimize mechanical degradation, and pilot-scale testing under variable water chemistries (pH, competing ions). Bridging these gaps will accelerate the translation of this technology from lab-scale innovation to real-world deployment, addressing global arsenic contamination with a resource-efficient, circular economy approach.

## 4. Materials and Methods

### 4.1. Synthesis of Magnetic-Functionalized Xerogels and Variation in the M/R Ratio

The RF gels in this study were prepared via a sol–gel polymerization process. Production of Fe_3_O_4_ NPs involved a co-precipitation method, based on procedures detailed by Hernández-Flores et al. (2018) [51].

Magnetic-functionalized carbon xerogels (XMCs) were synthesized using a sol–gel method with a fixed resorcinol-to-formaldehyde ratio of 0.5, along with constant resorcinol-to-catalyst (100) and resorcinol-to-water (0.04) ratios, using direct sonication [52]. The primary synthesis variable was the molar ratio of M/R, which was systematically adjusted to produce four distinct samples: XMC7 (M/R = 0.03), XMC8 (M/R = 0.04), XMC9 (M/R = 0.05), and XMC10 (M/R = 0.07). Direct sonication was employed during synthesis to ensure uniform dispersion of Fe_3_O_4_ NPs within the RF xerogel matrix. This controlled variation in M/R enabled systematic investigation of magnetite loading effects on the material’s physicochemical properties and adsorption performance.

After curing and drying according to the method described by Khamkure (2021) [53], the dried materials were ground with a porcelain mortar and sieved through a stainless-steel mesh sieve (125 µm). The resulting dried XMCs were black, polymer-derived magnetic xerogel materials.

### 4.2. Optimizing Conditions: The Role of Carbonization Temperature

The effect of carbonization temperatures (600–850 °C) on the preparation of magnetic-functionalized carbon xerogels was investigated to identify optimal synthesis conditions. The carbonization process was carried out in a GSL-1600X-50-UL tubular furnace (MTI Corporation, Richmond, VA, USA), which included a 50 mm alumina tube.

Carbonization was conducted with a heating rate of 2 °C/min and a nitrogen (N_2_) flow rate of 100 mL/min. The XMC10 sample, when pyrolyzed at 600 °C for 6 h, was designated XMC10-600. Furthermore, several samples underwent a stepwise pyrolysis regime, involving heating to 350 °C for 1 h, followed by heating to 850 °C for 2 h. After carbonization, the resulting magnetic xerogels were labeled to reflect their pyrolysis temperatures and applied treatments; examples include XD100-850, XMC7-850, XMC8-850, XMC9-850, XMC10-850, and XMC10-600.

### 4.3. Surface Modification Using H_2_O_2_ of Magnetic-Functionalized Carbon Xerogels

Surface modification with H_2_O_2_ was performed to enhance metal binding on the carbon xerogel surface, following a modified method based on Embaby et al. (2021) [54]. Selected samples (XMC10-600 and XMC10-850) underwent H_2_O_2_ treatment, resulting in modified materials labeled XMC10-600M and XMC10-850M.

A reflux system maintained the reaction temperature at 60 °C. A total of 50 mL of 30% hydrogen peroxide (H_2_O_2_, Jalmek) was added dropwise through a 125 mL standard taper stopper into a three-neck distillation flask containing 3 g of carbon xerogel. The H_2_O_2_ was slowly introduced by adjusting a stopcock until the reservoir was emptied. Throughout the 2 h process, the mixture was continuously stirred with a magnetic stir bar, and the temperature was kept constant at 60 °C.

Once the reaction was complete, the solution was cooled to room temperature. The modified carbon xerogel was recovered by filtration and extensively washed with 75 °C deionized water until the filtrate’s pH stabilized at approximately 7. The resulting product was then oven-dried at 100 °C for a duration of 15 h.

### 4.4. Characterization of Materials

XRD analysis was performed using an X’Pert Philips PW3040 diffractometer (Almelo, The Netherlands). The material was prepared for subsequent analysis by sieving it through a 200-mesh screen, which yielded a sample with an average particle size of 74 µm.

A dual-beam scanning electron microscope/focused ion beam (SEM/FIB) system (Helios NanoLab 600; Thermo Fisher Scientific, Hillsboro, OR, USA), coupled with energy-dispersive X-ray spectroscopy (EDS) and electron backscatter diffraction (EBSD), was used to analyze the surface texture and microstructure of Fe_3_O_4_ NPs. Magnetic-functionalized carbon xerogels were imaged at high resolution with a JEOL 7800F Prime field-emission scanning electron microscope (FE-SEM; Tokyo, Japan) operating at 5 keV. The chemical composition was determined with a environmental scanning electron microscope (ESEM) featuring EDS microanalysis.

Transmission electron microscopy (TEM) was performed using an FEI Talos F200S G2 microscope operating at 200 kV, equipped with three quantitative energy-dispersive X-ray spectroscopy (EDX) detectors to analyze chemical composition and nanoscale features (Fisher Scientific, Eindhoven, The Netherlands). High-resolution TEM (HR-TEM) imaging was employed to study composite structures at a resolution of 0.16 nm.

To identify surface functional groups, present in the synthesized materials, Fourier Transform Infrared (FTIR) spectroscopy was employed. Additionally, FTIR analysis was conducted on magnetic-functionalized carbon xerogels both before and after As(V) adsorption to elucidate the mechanism of arsenic ion uptake. Spectra were obtained using a Shimadzu IRAffinity-1S spectrometer (Shimadzu Corp., Kyoto, Japan) with dry powder samples, equipped with a Specac GS10800 attenuated total reflection (ATR) accessory featuring a type IIIa single-crystal diamond. All spectra were recorded across a wavenumber range of 400–4000 cm^−1^ with 45 scans per sample.

The specific surface area, adsorption/desorption profiles, and pore size distribution were characterized via an automated volumetric sorption analyzer (3P Instruments GmbH & Co. KG, Model BK100C; Odelzhausen, Germany). Measurements were conducted in an N_2_ atmosphere at 77 K. Before analysis, samples underwent degassing at 150 °C for a duration of 5 h. The surface area was calculated using the Brunauer–Emmett–Teller (BET) method, while the Barrett–Joyner–Halenda (BJH) method was used to determine pore size distribution [55].

The Fe content (weight percentage) of the synthesized magnetic-functionalized carbon xerogels was quantified using an atomic absorption spectrometer (Analyst 400, PerkinElmer, Shelton, CT, USA). The point of zero charge (pH_pzc_) was determined through potentiometric titrations [56].

### 4.5. The Kinetics of Arsenic Adsorption

The adsorption kinetics of arsenate (As(V)) were investigated using batch experiments with magnetic carbon xerogels XMC10-600M and XMC10-850M, pyrolyzed at 600 °C and 850 °C, respectively. A 1.024 mg/L As(V) solution was prepared, and 500 mg of adsorbent (2 g/L dosage) was added to 250 mL of the solution in Erlenmeyer flasks. The pH was adjusted to 3.00 using HCl/NaOH, and the mixtures were agitated at 150 rpm on an orbital shaker under ambient laboratory conditions (26 ± 1 °C). At predefined intervals (3, 6, 10, 30, 60, 120, 240, 360, 1200, 1320, and 1440 min), 5 mL aliquots were sampled, filtered through 0.45 μm pore-size membrane filters (Millipore, Burlington, MA, USA) using a vacuum filtration apparatus, and analyzed via an atomic absorption spectrometer (AAS) (Analyst 400; Perkin Elmer, Waltham, MA, USA).

The experimental data were evaluated using two key parameters: arsenic removal and adsorption capacity. The percentage of As(V) removal was calculated using the following Equation (2):

(2) $$\%removal=\frac{C_{0}-C_{e}}{C_{0}}\times 100$$

where *C*_0_ and *C_e_* represent the initial and equilibrium concentrations (mg/L) of arsenic in the aqueous solution, respectively. 

The adsorbed amount at time t (q_t_, mg/g) was calculated using the mass balance Equation (3),

q_t_ = [(C_o_ − C_t_) × V]/m (3)

where C_o_ and C_t_ are the initial and time-dependent concentrations (mg/L), V is the solution volume (L), and m is the adsorbent mass (g). The time-dependent adsorption data were analyzed using four nonlinear kinetic models to elucidate the governing mechanisms: Pseudo-First Order (PFO), Pseudo-Second Order (PSO), Elovich, and Power [46,57,58]. Nonlinear regression was performed using the statistical software R v4.2 to fit experimental data.

### 4.6. Determining Optimal Conditions for Arsenate Desorption Using Response Surface Methodology (RSM)

The preliminary desorption study aimed to evaluate the efficiency of various acidic and alkaline agents in recovering As(V) from saturated magnetic-functionalized carbon xerogels. The experiment utilized adsorbent material pre-loaded with As(V) at an initial concentration of 0.0896 mg/L. A fixed dose of 25 mg of the As-loaded adsorbent was introduced into 12.5 mL of each desorbing agent, including 0.10 M solutions of KOH, NaOH, HCl, HNO_3_, and CH_3_COOH. The mixtures were agitated at 150 rpm for 180 min to ensure equilibrium. Post-desorption, the supernatant was filtered, and arsenic concentration was analyzed by using inductively coupled plasma optical emission spectrometry (ICP-OES) (Model: Optima 8300, PerkinElmer, USA) to determine residual As(V) concentrations. Desorption efficiency was calculated as the percentage ratio of arsenic released to the initially adsorbed amount.

RSM utilizes advanced mathematical and statistical techniques to develop models that correlate a dependent variable (*Y*) with multiple independent variables (*x_i_*). RSM identifies optimal process conditions—specifically, the combination of variables (*x_i_*) that either maximizes or minimizes the response (*Y*). This approach employs sophisticated modeling using a second-order polynomial equation to establish the best-fit relationship between variables and outcomes, enabling precise process optimization. The second-order model is given by the following:

(4) $$Y=\beta_{0}+\sum_{i=1}^{k}\beta_{i}x_{i}+\sum_{i=1}^{k}\beta_{ii}x_{i}^{2}+\sum_{i=1<}^{k}\sum_{j=1}^{k}\beta_{ij}x_{i}x_{j}+\varepsilon$$

where *Y* is the predicted response variable, *β*_0_ is the constant coefficient (representing the y-intercept where the regression line crosses the y-axis), *β_i_* represents the linear coefficient (slope of the line), *b_ij_* denotes the interaction coefficient, *b_ii_* corresponds to the quadratic coefficient, *x_i_* and *x_j_* are the independent variables, and *ε* is the error term [48,59].

In this study, RSM was applied to determine the optimal conditions for arsenate. RSM was employed in this study to optimize conditions for arsenate desorption, investigating the effectiveness of two different desorption agents: potassium hydroxide (KOH) and nitric acid (HNO_3_). A three-factor design, incorporating desorbing concentration, adsorbent dose, and agitation speed, was used, with each variable tested at low, medium, and high levels (Table 3). The experimental design included two center points. The R statistical software package (version 4.0.3) was used for both data analysis and experimental design implementation.

### 4.7. Arsenic Adsorbent Regeneration

To regenerate arsenic-adsorbed XMC10-850M, a desorption experiment was conducted following an initial kinetic adsorption study. This adsorption phase was performed under optimized conditions (pH 3.0, 1440 min contact time, and an initial As(V) concentration of 1.024 mg/L) to ensure saturation of the adsorbent. The regeneration (desorption) phase then employed optimal desorption parameters, as determined through a separate RSM study.

### 4.8. Arsenic Quantification

The residual arsenic (As(V)) concentration in adsorption and desorption solutions was quantified using inductively coupled plasma optical emission spectrometry (ICP-OES, Optima 8300, PerkinElmer, Shelton, CT, USA). Samples were filtered through 0.45 μm nylon membranes to remove particulates and acidified with 2% HNO_3_ (*v/v)* to stabilize As(V). This quantitative analysis enabled the calculation of both the arsenic adsorption efficiency (based on the decrease in concentration after contact with the adsorbent) and the arsenic desorption efficiency (based on the concentration released into the regeneration solution).

## Figures and Tables

**Table 1:** Pore Volume Distribution of XMC10-850M.

Pore Size Range (nm)	Pore Volume (cm^3^/g)	Percentage (%)
2.00–10.00	0.13751	15.22
10.00–20.00	0.09271	10.26
20.00–50.00	0.18482	20.45
>50.00	0.48856	54.07

**Table 2:** Estimation of the kinetic model parameters and correlation coefficients for As(V) adsorption by magnetic-functionalized carbon xerogels XMC10-850M.

Kinetic Models	Parameters and Correlation Coefficients	XMC10-600M	XMC10-850M
Pseudo-First-Order equation (PFO)	k_1_	0.2837	0.0046
	q_t_ (µg/g)	211.23	459.4
	R^2^	0.218	0.846
	RMSE	45.73	65.23
Pseudo-Second-Order equation (PSO)	k_2_	0.0018	0.00001
	q_t_ (µg/g)	220.9	497.7
	R^2^	0.3319	0.8977
	RMSE	42.29	53.25
Elovich	β	0.0476	0.0102
	α	2795.3	8.048
	R^2^	0.7375	0.9396
	RMSE	26.51	40.90
Power	β	0.1190	0.3483
	α	111.5	39.07
	R^2^	0.8037	0.9443
	RMSE	22.92	39.30

**Table 3:** RSM variable configuration to optimize As(V) desorption using KOH and HNO3 as a desorbing agent.

Factors	Coding Factors	Desorbing Agent					
		HNO_3_			KOH		
		Low	Center	High	Low	Center	High
		(−1)	0	−1	(−1)	0	−1
Desorbing solution concentration (M)	*x*_1_	0.05	0.15	0.25	0.5	1	1.5
Agitation speed (rpm)	*x*_2_	80	120	160	80	120	160
Dose of used adsorbent (g/L)	*x*_3_	0.4	1.2	2	0.4	1.2	2

## Data Availability

Data are contained within the article.

## References

[1] Valskys V., Hassan H.R., Wołkowicz S., Satkūnas J., Kibirkštis G., Ignatavičius G. (2022). A Review on Detection Techniques, Health Hazards and Human Health Risk Assessment of Arsenic Pollution in Soil and Groundwater. Minerals.

[2] Alka S., Shahir S., Ibrahim N., Ndejiko M.J., Vo D.V.N., Manan F.A. (2021). Arsenic Removal Technologies and Future Trends: A Mini Review. J. Clean Prod..

[3] Indah S., Helard D., Binuwara A. (2017). Studies on Desorption and Regeneration of Natural Pumice for Iron Removal from Aqueous Solution. Water Sci. Technol..

[4] Ccamerccoa M.H., Falcon N.L.T., Félix L.L., Pacheco-Salazar D.G., Aragón F.F.H., Coaquira J.A.H., Garnier J., Vera-Gonzales C. (2022). High Efficiency of Magnetite Nanoparticles for the Arsenic Removal from an Aqueous Solution and Natural Water Taken from Tambo River in Peru. J. Environ. Health Sci. Eng..

[5] Nisticò R. (2021). A Synthetic Guide toward the Tailored Production of Magnetic Iron Oxide Nanoparticles. Bol. Soc. Esp. Ceram. Y Vidr..

[6] Dube E., Okuthe G.E. (2023). Engineered Nanoparticles in Aquatic Systems: Toxicity and Mechanism of Toxicity in Fish. Emerg. Contam..

[7] Gambardella C., Pinsino A. (2022). Nanomaterial Ecotoxicology in the Terrestrial and Aquatic Environment: A Systematic Review. Toxics.

[8] Awadallah-F A., Al-Muhtaseb S.A. (2017). Novel Controlled Synthesis of Nanoporous Carbon Nanorods from Resorcinol-Formaldehyde Xerogels. Mater. Lett..

[9] Morales-Torres S., Jirglová H., Pastrana-Martínez L.M., Maldonado-Hódar F.J. (2020). Influence of Electrostatic Interactions during the Resorcinol-Formaldehyde Polymerization on the Characteristics of Mo-Doped Carbon Gels. Processes.

[10] Han Y., Zhang L., Yang W. (2024). Synthesis of Mesoporous Silica Using the Sol–Gel Approach: Adjusting Architecture and Composition for Novel Applications. Nanomaterials.

[11] Veselov G.B., Vedyagin A.A. (2023). Resorcinol–Formaldehyde-Derived Carbon Xerogels: Preparation, Functionalization, and Application Aspects. Materials.

[12] Al-Muhtaseb S.A., Ritter J.A. (2003). Preparation and Properties of Resorcinol-Formaldehyde Organic and Carbon Gels. Adv. Mater..

[13] Muthu Prabhu S., Rane N.R., Li X., Otari S.V., Girawale S.D., Palake A.R., Kodam K.M., Park Y.K., Ha Y.H., Kumar Yadav K. (2023). Magnetic Nanostructured Adsorbents for Water Treatment: Structure-Property Relationships, Chemistry of Interactions, and Lab-to-Industry Integration. Chem. Eng. J..

[14] Peralta M.E., Ocampo S., Funes I.G., Medina F.O., Parolo M.E., Carlos L. (2020). Nanomaterials with Tailored Magnetic Properties as Adsorbents of Organic Pollutants from Wastewaters. Inorganics.

[15] Mudhoo A., Sillanpää M. (2021). Magnetic Nanoadsorbents for Micropollutant Removal in Real Water Treatment: A Review. Environ. Chem. Lett..

[16] Perez Mora B., Bellú S., Mangiameli M.F., Frascaroli M.I., González J.C. (2019). Response Surface Methodology and Optimization of Arsenic Continuous Sorption Process from Contaminated Water Using Chitosan. J. Water Process Eng..

[17] Sahu U.K., Mahapatra S.S., Patel R.K. (2018). Application of Box–Behnken Design in Response Surface Methodology for Adsorptive Removal of Arsenic from Aqueous Solution Using CeO2/Fe2O3/Graphene Nanocomposite. Mater. Chem. Phys..

[18] Gugushe A.S., Nqombolo A., Nomngongo P.N. (2019). Application of Response Surface Methodology and Desirability Function in the Optimization of Adsorptive Remediation of Arsenic from Acid Mine Drainage Using Magnetic Nanocomposite: Equilibrium Studies and Application to Real Samples. Molecules.

[19] Ortiz Letechipia J., González-Trinidad J., Júnez-Ferreira H.E., Bautista-Capetillo C., Robles-Rovelo C.O., Contreras Rodríguez A.R., Dávila-Hernández S. (2022). Aqueous Arsenic Speciation with Hydrogeochemical Modeling and Correlation with Fluorine in Groundwater in a Semiarid Region of Mexico. Water.

[20] Rahidul Hassan H. (2023). A Review on Different Arsenic Removal Techniques Used for Decontamination of Drinking Water. Environ. Pollut. Bioavailab..

[21] Devrajani S.K., Ahmed Z., Qambrani N.A., Kanwal S., Sundaram U.M., Mubarak N.M. (2024). Mechanism of Arsenic Removal Using Brown Seaweed Derived Impregnated with Iron Oxide Biochar for Batch and Column Studies. Sci. Rep..

[22] Zhang Y., Huang K. (2019). Defluoridation Behavior of Layered Fe-Mg-Zr Hydroxides and Its Continuous Purification of Groundwater. Colloids Surf. A Physicochem. Eng. Asp..

[23] Santoyo-Cisneros R., Rangel-Mendez J.R., Nava J.L., Larios-Durán E.R., Chazaro-Ruiz L.F. (2020). Influence of Surface Chemistry of Activated Carbon Electrodes on Electro-Assisted Adsorption of Arsenate. J. Hazard. Mater..

[24] Faizal A.N.M., Zaini M.A.A. (2019). Resorcinol-Formaldehyde Carbon Gels Adsorption: A Commentary. Acta Chem. Iasi.

[25] Cao X., Xiao F., Xie X., Li X., Li G., Li L., Zhang Q., Zhang W., You X., Gai Y. (2021). Adsorption and Desorption of Hg(II) from Aqueous Solution Using Magnetic Fe3O4@PPy Composite Microspheres. J. Water Reuse Desalination.

[26] Ptaszkowska-Koniarz M., Goscianska J., Bazan-Wozniak A., Pietrzak R. (2022). Amine-Modified Carbon Xerogels as Effective Carbon-Based Adsorbents of Anionic Dye from Aqueous Solutions. Materials.

[27] Schwaminger S.P., Bauer D., Fraga-García P., Wagner F.E., Berensmeier S. (2017). Oxidation of Magnetite Nanoparticles: Impact on Surface and Crystal Properties. CrystEngComm.

[28] Zaefferer S., Habler G. (2017). Scanning Electron Microscopy and Electron Backscatter Diffraction. Eur. Mineral. Union. Notes Mineral..

[29] de Jesús Ruíz-Baltazar Á., Reyes-López S.Y., de Lourdes Mondragón-Sánchez M., Robles-Cortés A.I., Pérez R. (2019). Eco-Friendly Synthesis of Fe_3_O_4_ Nanoparticles: Evaluation of Their Catalytic Activity in Methylene Blue Degradation by Kinetic Adsorption Models. Results Phys..

[30] Habibi N. (2015). Functional Biocompatible Magnetite-Cellulose Nanocomposite Fibrous Networks: Characterization by Fourier Transformed Infrared Spectroscopy, X-Ray Powder Diffraction and Field Emission Scanning Electron Microscopy Analysis. Spectrochim. Acta A Mol. Biomol. Spectrosc..

[31] Elashmawi I.S., Alhusaiki-Alghamdi H.M. (2024). Fabrication, Characterization, Spectroscopic, and Magnetic Properties of Polyaniline/Magnetite (PANi/Fe_3_O_4_) Nanocomposites. Opt. Quantum Electron..

[32] Rodewald J., Thien J., Ruwisch K., Pohlmann T., Hoppe M., Schmalhorst J., Küpper K., Wollschläger J. (2024). Structure-Related Electronic and Magnetic Properties in Ultrathin Epitaxial NixFe3−xO4 Films on MgO(001). Nanomaterials.

[33] Sikora P., Horszczaruk E., Cendrowski K., Mijowska E. (2016). The Influence of Nano-Fe3O4 on the Microstructure and Mechanical Properties of Cementitious Composites. Nanoscale Res. Lett..

[34] Yi S., Liu J., Wang C., Miao P., Liang J., Wang X. (2020). Effects of Carbonization Temperature on Structure and Mechanical Strength of Electrospun Carbon Nanofibrous Mats. Mater. Lett..

[35] Khamkure S., Bustos-Terrones V., Torrecilla-Valle A., Gamero-Melo P., Reyes-Rosas A., Vargas-Gutiérrez G., Garrido-Hoyos S.E. (2023). Optimization Study for Desorption of Arsenic and Regeneration Performance on Magnetic Carbon Xerogels for Environmental Sustainability †. Eng. Proc..

[36] Das A., Hansda K.M., Mahata N. (2020). Tuning of Pore Texture of Carbon Xerogels Synthesized Using Resorcinol and Paraformaldehyde as Precursors. J. Indian. Chem. Soc..

[37] Ilyas S., Abdullah B., Tahir D. (2019). X-Ray Diffraction Analysis of Nanocomposite Fe3O4/Activated Carbon by Williamson–Hall and Size-Strain Plot Methods. Nano-Struct. Nano-Objects.

[38] Sun T., Levin B.D.A., Schmidt M.P., Guzman J.J.L., Enders A., Martínez C.E., Muller D.A., Angenent L.T., Lehmann J. (2018). Simultaneous Quantification of Electron Transfer by Carbon Matrices and Functional Groups in Pyrogenic Carbon. Environ. Sci. Technol..

[39] Attia S.M., Abdelfatah M.S., Mossad M.M. (2017). Conduction Mechanism and Dielectric Properties of Pure and Composite Resorcinol Formaldehyde Aerogels Doped with Silver. J. Phys. Conf. Ser..

[40] Gore P., Khraisheh M., Kandasubramanian B. (2018). Nanofibers of Resorcinol–Formaldehyde for Effective Adsorption of As (III) Ions from Mimicked Effluents. Environ. Sci. Pollut. Res..

[41] Min X., Li Y., Ke Y., Shi M., Chai L., Xue K. (2017). Fe-FeS2 Adsorbent Prepared with Iron Powder and Pyrite by Facile Ball Milling and Its Application for Arsenic Removal. Water Sci. Technol..

[42] Ruiz-Mora M.S., Alfaro-Cuevas-Villanueva R., Martínez-Miranda V., Hernández-Cristóbal O., Cortés-Martínez R. (2022). Removal of As(V) from Aqueous Solutions Using Calcium-Alginate Microspheres with Encapsulated Iron Nanoparticles. Water Supply.

[43] Sahu U.K., Mandal S., Sahu S., Gouda N., Patel R.K. (2022). Preparation and Characterization of Mesoporous Cerium Oxide for Toxic As(V) Removal: Performance and Mechanistic Studie. J. Environ. Eng. Landsc. Manag..

[44] Hao L., Liu M., Wang N., Li G. (2018). A Critical Review on Arsenic Removal from Water Using Iron-Based Adsorbents. RSC Adv..

[45] Malaika A., Morawa Eblagon K., Soares O.S.G.P., Pereira M.F.R., Figueiredo J.L. (2020). The Impact of Surface Chemistry of Carbon Xerogels on Their Performance in Phenol Removal from Wastewaters via Combined Adsorption-Catalytic Process. Appl. Surf. Sci..

[46] Zhu H., Lin W., Fan L. (2023). Novel Method for the Arsenic Removal Experiment and Mechanism Analysis. ACS Omega.

[47] Antić K., Onjia A., Vasiljević-Radović D., Veličković Z., Tomić S.L. (2021). Removal of Nickel Ions from Aqueous Solutions by 2-Hydroxyethyl Acrylate/Itaconic Acid Hydrogels Optimized with Response Surface Methodology. Gels.

[48] Bayuo J., Rwiza M.J., Choi J.W., Sillanpää M., Mtei K.M. (2024). Optimization of Desorption Parameters Using Response Surface Methodology for Enhanced Recovery of Arsenic from Spent Reclaimable Activated Carbon: Eco-Friendly and Sorbent Sustainability Approach. Ecotoxicol. Environ. Saf..

[49] Tabatabaei F.S., Izanloo H., Heidari H., Vaezi N., Zamanzadeh M., Nadali A., Aali R., Asadi-Ghalhari M. (2020). Modeling and Optimization of Arsenic (III) Removal from Aqueous Solutions by GFO Using Response Surface Methodology. Pollution.

[50] Olivares C., Reis F.D.A.A. (2019). Interplay of Adsorption and Surface Mobility in Tracer Diffusion in Porous Media. Phys. Rev. E.

[51] Hernández-Flores H., Pariona N., Herrera-Trejo M., Hdz-García H.M., Mtz-Enriquez A.I. (2018). Concrete/Maghemite Nanocomposites as Novel Adsorbents for Arsenic Removal. J. Mol. Struct..

[52] Khamkure S., Gamero-Melo P., Garrido-Hoyos S.E., Reyes-Rosas A., Pacheco-Catalán D.E., López-Martínez A.M. (2023). The Development of Fe3O4-Monolithic Resorcinol-Formaldehyde Carbon Xerogels Using Ultrasonic-Assisted Synthesis for Arsenic Removal of Drinking Water. Gels.

[53] Khamkure S., Garrido-Hoyos S.E., Gamero-Melo P., Reyes-Rosas A. (2021). Synthesis and Characterization of Magnetic Xerogel Monolith as an Adsorbent for As(V) Removal from Groundwater. Processes.

[54] Embaby M.A., Abdel Moniem S.M., Fathy N.A., El-kady A.A. (2021). Nanocarbon Hybrid for Simultaneous Removal of Arsenic, Iron and Manganese Ions from Aqueous Solutions. Heliyon.

[55] Verma N.K., Khare P., Verma N. (2015). Synthesis of Iron-Doped Resorcinol Formaldehyde-Based Aerogels for the Removal of Cr(VI) from Water. Green. Process. Synth..

[56] Rajput S., Pittman C.U., Mohan D. (2016). Magnetic Magnetite (Fe_3_O_4_) Nanoparticle Synthesis and Applications for Lead (Pb2+) and Chromium (Cr6+) Removal from Water. J. Colloid. Interface Sci..

[57] Barbero G., Scarfone A.M., Evangelista L.R. (2022). The Kinetics of Sorption–Desorption Phenomena: Local and Non-Local Kinetic Equations. Molecules.

[58] López-Luna J., Ramírez-Montes L.E., Martinez-Vargas S., Martínez A.I., Mijangos-Ricardez O.F., González-Chávez M.D.C.A., Carrillo-González R., Solís-Domínguez F.A., Cuevas-Díaz M.d.C., Vázquez-Hipólito V. (2019). Linear and Nonlinear Kinetic and Isotherm Adsorption Models for Arsenic Removal by Manganese Ferrite Nanoparticles. SN Appl. Sci..

[59] Fard L.A., Salimi L., Nabi-Bidhendi G., Gafourian H., Baghdadi M. (2021). Removal of Cadmium Ions from Aqueous Solutions Using Nickel Metal-Organic Framework: Isotherm, Kinetic Studies, Optimization, and Modeling by Response Surface Methodology (RSM). Desalination Water Treat..

